# Transforming MRCPsych theory examinations: digitisation and very short answer questions (VSAQs)

**DOI:** 10.1192/bjb.2021.23

**Published:** 2022-02

**Authors:** Karl Scheeres, Niruj Agrawal, Stephanie Ewen, Ian Hall

**Affiliations:** 1 Centre for Health Sciences Education, University of Bristol, UK; 2 St George's Hospital, UK; 3 St George's, University of London, UK; 4 South London and Maudsley NHS Foundation Trust, UK; 5 East London NHS Foundation Trust, UK

**Keywords:** Education and training, supervision, information technologies, cost-effectiveness, history of psychiatry

## Abstract

Many examinations are now delivered online using digital formats, the migration to which has been accelerated by the COVID-19 pandemic. The MRCPsych theory examinations have been delivered in this way since Autumn 2020. The multiple choice question formats currently in use are highly reliable, but other formats enabled by the digital platform, such as very short answer questions (VSAQs), may promote deeper learning. Trainees often ask for a focus on core knowledge, and the absence of cueing with VSAQs could help achieve this. This paper describes the background and evidence base for VSAQs, and how they might be introduced. Any new question formats would be thoroughly piloted before appearing in the examinations and are likely to have a phased introduction alongside existing formats.

Examinations are now being delivered on online platforms in many undergraduate and postgraduate contexts. The COVID-19 pandemic has accelerated this, as digital platforms have the potential to enable examination delivery during lockdowns, or if trainees are isolating or in quarantine, without social distancing concerns. Education is also becoming increasingly international, and the MRCPsych examination is both sought after and has been delivered in international centres for many years. However, travel to examination centres for both staff and candidates is expensive, and significantly increases its overall carbon footprint.

The Royal College of Psychiatrists has therefore decided to deliver its theory examinations via digital platforms as from Autumn 2020, using a combination of artificial intelligence and in-person online proctoring (equivalent to traditional invigilators) to ensure that high standards of probity are maintained. The examinations will initially be delivered using the two existing question formats, multiple choice questions (MCQs) and extended matching questions (EMQs). However, digital platforms enable the use of new question formats that may allow more comprehensive coverage of the syllabus (the syllabus can be found at: https://www.rcpsych.ac.uk/training/exams/preparing-for-exams). We know that assessment has a powerful effect in driving learning,^1^ and multiple choice question formats may encourage rote learning from question banks. We will thoroughly evaluate any new question formats before we introduce them into the MRCPsych examination, but we would hope that they would encourage deeper and more holistic learning strategies that would better equip our future psychiatrists to have the biggest impact on the mental health of their patients.

## Choosing examination formats for the MRCPsych

When setting an examination, some of the key factors^2^ that need to be considered when assessing its utility are shown in Table 1. Each of these factors have to be weighed up against each other, with differing weightings according to the purpose and type of assessment.

**Table 1:** Key factors to be considered when assessing the utility of an assessment (adapted with permission from reference^2^)

Factor	Questions asked
Validity	Does the examination test what we want it to test?
Reliability	Are the results repeatable and accurate? Are external sources of error other than candidate ability accounted for and reduced?
Educational impact	What is the impact of the examination upon trainees’ learning? Does it lead to deeper learning and long-term retention?
Acceptability	Is the examination acceptable to those sitting it and other stakeholders?
Cost	Are costs reasonable?

MCQs are a format that lends itself to reliability through standardisation of answers and ease of evaluation of large numbers of candidates via machine marking. Although MCQs have been used since the inception of the MRCPsych in 1972, historically, short answer questions (SAQs) and essays were also utilised; these were phased out as individual marking of SAQs with increasing numbers of candidates was taxing, and there were questions about the reliability of essay marking.^3^ The format of MCQs evolved from initial true/false answers to the single best answer or ‘best of five’ in use today, as well as the use of the EMQs, in which there is a theme, several stems and a greater number of options, more easily assessing the application of knowledge.^4^ The MRCPsych is a high-stakes examination, with important consequences for candidates, our patients and society in general. In common with all high-stakes postgraduate medical assessments based in the UK, it is regulated closely by the General Medical Council, and all changes to format and structure must undergo prospective approval by them.

Given these concerns, the reliability of the MRCPsych must be extremely high, so that no trainee passes without the requisite ability. Fortunately, the written papers have excellent reliability (with Cronbach's alpha, a measure of reliability, consistently >0.85), but some have questioned whether this has happened at the expense of validity.^5,6^ Has the depth of clinical context and its application been lost? Perhaps we fail to reward those trainees who undertake in-depth study of complex issues, such as aetiology, ethics and the history of psychiatry.^5^ The main criticism of MCQs is a ‘cueing’ effect, whereby candidates are cued by the correct answer, rather than actively recalling it.^7^ There is evidence that requiring candidates to *construct* an answer, such as in SAQs, produces better memory than tests that require recognition.^8^ Additional issues with MCQs may include various ‘test-taking’ behaviours, such as eliminating wrong answers to arrive at the correct one, guessing from the options available and seeking clues from the language used to deduce the correct answer independently from the knowledge required.^9^

MCQs end up testing recognition memory, and recall is significantly affected by this cueing effect. Creating a good MCQ with valid and meaningful distractors (incorrect options) can be extremely hard. Poor-quality distractors can make guessing more rewarding. There are a number of areas of the syllabus where it is impossible to write valid distractors, and as a consequence, clinically meaningful knowledge may not be examined and more obscure areas, where MCQs may be easier to write, are more likely to be tested.

As mentioned above, it is intuitive and commonly recognised that the assessment drives learning.^1^ Areas of the syllabus that are more commonly examined are therefore more likely to be studied by trainees. Assessment factors contribute to strategies used to study,^10^ which could influence the trainees’ overall learning and the extent of knowledge achieved. Developments in technology have allowed easy access to online MCQ ‘question banks’. Many trainees therefore focus their effort on practicing these questions, rather than focusing on core learning and developing deeper understanding.

The costs of taking the MRCPsych for candidates are high^11^ because of the high cost of the infrastructure behind the examination, e.g. the professional examinations team, detailed psychometric analysis, and supporting the psychiatrists who volunteer their time freely to create and quality-assure questions, and analyse the results. For several years now, the examination is budgeted not to make excessive surpluses, but if this inadvertently happens, the surplus is directed to the trainees’ fund, which has previously funded the creation of the Trainees Online learning resource, among other projects. Moving to digital platforms may reduce costs to trainees as they no longer need travel or accommodation, and potentially could reduce overall costs as no physical venues are required; however, this is uncertain, and the costs of commercial contracts for software, training and ongoing IT support may counteract this.

## Digitisation of examinations

The COVID-19 pandemic led to a rapid and unpredicted introduction of online examinations for the MRCPsych, although the College had plans to begin moving toward digitisation before the pandemic. Although there is a relative paucity of literature on online examinations,^12^ one small study, in which a direct comparison of online examinations versus paper examinations was made, showed equivalent reliability and validity.^13^ In terms of candidate performance in online versus paper examinations, the few studies directly testing this have shown no significant differences.^13,14^ Candidates’ perception of online examinations are often favourable, and one study found reduced anxiety when taking online compared with traditional paper-based examinations.^14^ Possibly, the fact that candidates are not able to see their peers might account for this. However, it is clear that the rapid introduction of digitisation for the MRCPsych caused considerable anxiety in trainees; the same study^14^ recognised that the first sitting of online examinations can cause anxiety, which later subsides with familiarity upon repeated testing.

## Very short answer questions

Very short answer questions (VSAQs) are a novel format of written questions.^15–19^ A VSAQ consists of a short question for which an answer is required to be manually entered on computer screen from free recall, as open text. There are no options provided to choose from as in MCQs/EMQs. Generally, the answer would be only a few words. Box 1 shows some examples of how VSAQs may look. Any correct response will attract one mark and any incorrect response will attract zero marks. Examination software would be programmed to recognise multiple versions of correct answers, using smart algorithms. These would allow different versions of a correct response to be recognised. For example, the first question in Box 1 provides an example of several possible correct answers for that question; all of these answers would attract a full mark, and centre around the idea of a reduction or suppression of the default mode network. The software would additionally be programmed to highlight any answer that is a non-exact match (approximate) to any possible correct answers, and these will be manually reviewed by a designated and trained examiner to ascertain whether that represents a correct response. This will ensure that any unforeseen versions of correct responses will not go unrecognised and unrewarded. That response will then be saved in the list of correct answers for that question for any future examinations. Examiners will also review all other marking done by the computer, to ensure accuracy. Minor spelling errors or typos (e.g. ‘inihbited’ rather than ‘inhibited’) will not be penalised and will be picked up during the review process. VSAQs also allow for two entirely different but correct answers, as illustrated in the second example in Box 1. In this example, again, either of the responses will attract a full mark.

Box 1 Very short answer question examples.

Example 1: A very short answer question with different versions of the correct answer:

How does the ‘default mode network’ react in a healthy brain when one performs a goal-directed task?

Correct answers may include, but are not limited to:

- Decreased activity
- Reduced activity
- Inhibited
- Suppressed
- Switched off

Example 2: A very short answer question with different correct answers:

Name the neurotransmitter mechanism thought to be responsible for clozapine-induced hypersalivation.

Correct answers would include:

- Alpha 2 receptor antagonism
- Muscarinic M4 agonism

Again, differing versions of these correct answers would be accepted, e.g. a2 adrenergic antagonism.

The free recall tested by the VSAQs can be more easily focused on clinically relevant topics, and allow freedom to assess a wider spectrum of the syllabus where MCQs may be impossible to write. This should encourage trainees to refocus on core learning through textbooks and primary papers, and make their knowledge base more clinically relevant in the long term.

In the studies to date, VSAQs have been shown to have higher reliability than MCQs, and reduce the cueing effect.^15–17^ They may improve validity by testing nascent knowledge and clinical skills, rather than the ability to pass examinations.^15^ In one study of 300 medical students,^15^ 69% of students undertaking VSAQs felt that they were more representative of how they would be expected to answer questions in actual clinical practice, and about half felt that they would change their learning strategies in response. However, these studies were conducted on undergraduate medical students and may not be generalisable to postgraduate psychiatry trainees. Additionally, as far as we are aware, there has not been any published data that uses VSAQs from a high-stakes examination such as the MRCPsych, although at least one other College are considering their introduction for UK medical trainees.^20^ Finally, as VSAQs require recall rather than recognition, candidates appear to universally score lower in them when compared with MCQs;^15–19^ this must be carefully accounted for in the standard setting process that sets the pass mark, so that standard setting judges are aware of likely lower scores in comparison with MCQs, particularly in first iterations of the test when they are lacking comparative past data. To account for this, there would be pilot questions tested and a full analysis undertaken to inform future standard setting.

## Trainees’ views on digitisation and VSAQs

The opinion of psychiatry trainees was obtained via a presentation by the Chief Examiner, Dr Ian Hall, to the Psychiatric Trainees’ Committee. The Examinations Sub-Committee's Trainee Representative also sought feedback on the Psychiatric Trainees’ Committee collaborative platform, ‘Workplace’. The questions submitted to the College's webinar, ‘MRCPsych Exam – Changes to exam delivery this Autumn’, attended by over a thousand psychiatry trainees and supervisors, were also reviewed in summarising concerns with regards to the digitisation of the theory examinations.

Psychiatry trainees raised several concerns with regards to the digitisation of the theory examinations (Table 2). In the context of sitting the examinations from home, a common theme was how technical issues, such as insufficient internet connectivity, would be resolved, what support would be available to assist with this, and how the College would ensure candidates were not disadvantaged as a result of technical issues. Trainees also expressed concerns as to how cheating would be identified, particularly the potential to ‘trick’ proctoring technology, to prevent inflated examination marks disadvantaging other trainees. Similarly, they expressed concerns that trainees may be falsely accused of cheating if they write notes or look away from the screen. The concerns regarding cheating are in keeping with the published literature of both candidates’ and examination setters’ perceptions of online examinations.^12^ Trainees also noted that some trainees’ home environments may be unsuitable for sitting examinations, because of caring commitments or house-sharing arrangements. Trainees were also keen to understand how candidates with dyslexia and other specific learning needs would be accommodated. Furthermore, trainees expressed an expectation that examination fees would be reduced in the context of digital examinations.

**Table 2:** Common themes of trainees’ concerns and responses

Concern	Reponses
Technical issues, e.g. internet connectivity	The College partners with third-party software providers who have both expertise and a track record in high-stakes online examination delivery. Trainees are encouraged to test the resilience of their internet and device in advance, using provided software. Software developers design software to account for brief interruptions, and protocols exist for more significant technical issues.
Cheating, proctoring and false accusations	All alerts from the artificial intelligence software proctoring are reviewed by a live proctor. Final decisions about cheating are made following rigorous review by the Examinations Sub-committee, and subject to the normal appeals process.
Unsuitable home environment	Candidates can choose any suitable workstation with reliable internet to take the examination, e.g. a family member's or friend's house, a work or university computer.
Examination should not be reduced to a ‘spelling test’ in very short answer questions	Variations in answers and spelling mistakes will be accounted for, and examiners would review incorrect answers, including typos and spelling errors.

Despite the concerns raised, trainees generally appeared to agree with the prospect of the digitisation of the theory examinations, even outside the current context of COVID-19. However, many expressed a strong preference for these to be conducted in test centres to prevent technical issues or cheating, and to ensure candidates with home settings unsuitable for sitting examinations were not disadvantaged.

With regards to the introduction of VSAQs, the trainee response was generally positive. Trainees felt it addressed their request for a greater emphasis on the testing of core knowledge and that VSAQs were better at testing the application of knowledge than the current format. However strong concerns were raised with regards to the examinations not becoming a ‘spelling test’, and particularly that this may disadvantage candidates with dyslexia, other specific learning needs and international medical graduates. They noted that not all spelling errors are of equal clinical significance and where it is clear that a candidate's intended meaning is correct, that this should be accepted as a correct answer.

## Conclusions and future directions

The digitisation of examinations is inevitable, and the pace of change has been rapid as a result of the COVID-19 pandemic. For the MRCPsych theory papers, this could bring several improvements in terms of examination delivery, such as improved convenience and access to the examination, and faster processing of results. However, it also brings opportunities for improving assessment. We hope that a careful, phased introduction of alternative question formats such as VSAQs will enable a more comprehensive sampling of the examination syllabus, a greater focus on core knowledge and promote deeper, more holistic and integrated learning strategies. We know that these issues are of importance to trainees and clinical educators alike.

Any change like this requires comprehensive evaluation and testing, and because this is a high-stakes postgraduate medical qualification, the UK General Medical Council will need to prospectively approve any changes.^21^ As mentioned above, before any partial introduction, we plan to pilot questions on trainees and conduct an extensive psychometric analysis of the results. This would include an equality analysis to assess the impact on differential attainment in protected groups. The successful delivery of such a change requires comprehensive stakeholder engagement, and none are more important that the doctors training in psychiatry who take the examination; we plan ongoing consultation with trainees. We must also ensure that our training programmes prepare candidates thoroughly, with supervisors and tutors being up to date with new assessment methodologies and the reasons for their introduction. There would be the potential for online learning platforms to assist trainees with the new style questions. Stakeholder feedback has been largely positive on the face validity of VSAQs, in promoting the acquisition of knowledge that will be useful in clinical practice, and so help deliver better healthcare for people with mental health problems.

## About the authors

**Karl Scheeres** is a lecturerat the Centre for Health Sciences Education at the University of Bristol, UK, and Chair of Standard Setting for MRCPsych theory papers at the Royal College of Psychiatrists, UK. **Niruj Agrawal** is Lead Consultant Neuropsychiatrist at St George's Hospital, UK, and an honorary senior lecturer at St George's, University of London, UK. He is also Lead for VSAQs for MRCPsych examinations at the Royal College of Psychiatrists, UK. **Stephanie Ewen** is a specialist registrar in psychiatry of intellectual disability at South London and Maudsley NHS Foundation Trust, UK, and the Trainee Representative on the Royal College of Psychiatrists Examinations Sub-Committee, UK. **Ian Hall** is a consultant psychiatrist for people with intellectual disabilities at East London NHS Foundation Trust, UK, and Chief Examiner at the Royal College of Psychiatrists, UK.
